# Cell Division Cycle-Associated Genes Are Potential Immune Regulators in Nasopharyngeal Carcinoma

**DOI:** 10.3389/fonc.2022.779175

**Published:** 2022-02-14

**Authors:** Danxian Jiang, Yin Li, Jinxin Cao, Lianghe Sheng, Xinhai Zhu, Meng Xu

**Affiliations:** ^1^ Department of Oncology, The First Affiliated Hospital of Jinan University, Jinan University, Guangzhou, China; ^2^ Department of Head and Neck Oncology, Affiliated Hospital of Guangdong Medical University, Zhanjiang, China

**Keywords:** cell division cycle-associated gene family, CDCAs, nasopharyngeal carcinoma, NPC, immune, prognosis

## Abstract

**Background:**

Cell division cycle-associated (CDCA) gene family is essential to cell cycle regulation. Numerous studies have illuminated that dysfunction of CDCA genes may not only lead to uncontrolled cell proliferation resulting in tumorigenesis but also influence immune cell infiltration in tumors. However, the role of the CDCA gene family on the prognosis and immune infiltration in nasopharyngeal carcinoma (NPC) remains to be unclear.

**Methods:**

SBC human ceRNA array V1.0 was used to measure mRNA expression in three pairs of NPC tissues and nasopharyngitis tissues. The expression of CDCA8 was confirmed in an IHC microarray containing 130 NPC patients. Two external GEO cohorts were enrolled for further analysis. Prognosis analysis was performed using the Kaplan–Meier method. Gene set enrichment analysis (GSEA) was applied to explore the potential mechanism of CDCA genes in NPC. The relationship between CDCA gene family and immune infiltration in NPC was evaluated using the Xcell tool.

**Results:**

CDCA genes were broadly upregulated in NPC tissues compared to nasopharyngitis tissues, and high expression of CDCA3/5/8 indicated worse prognosis in NPC. Besides cell cycle pathways, we found that CDCA3/5/8 were involved in multiple immune-related pathways. Overexpression of CDCA8 was strongly associated with less infiltration of CD8+ T cells and more infiltration of CD4+ Th1 cells and was negatively correlated with immune checkpoint blockade (ICB)-related genes.

**Conclusion:**

CDCA gene family was upregulated in NPC, and their expressions were associated with adverse prognosis. High expression of CDCA8 was associated not only with poor prognosis, but also with less immune infiltration and downregulation of ICB-related genes in NPC.

## Introduction

Nasopharyngeal carcinoma (NPC) is a squamous cell carcinoma arising from the epithelial lining of the nasopharynx with a specific geographic distribution. As reported by the International Agency for Research on Cancer, there were over 130,000 new cases of NPC in 2020 and more than 70% of new cases are reported in East and Southeast Asia (1). In China, the annual incidence of NPC is about 30 cases per 100,000 persons in southern China (2). Approximately 70% of newly diagnosed NPC patients are at advanced stages. Intensity-modulated radiation therapy (IMRT) is the recommended therapy for advanced NPC patients (3), but patients are more likely to suffer treatment failure from radiotherapy due to radioresistance, which is the leading cause of NPC mortality (4). NPC patients in the late stages treating with IMRT usually suffer from severe treatment-related complications that reduce the quality of life (5). Recently, many studies have demonstrated the effectiveness of immunotherapy in EBV-associated NPC (6). Some trials are working on evaluating the value of immunotherapy in NPC (7), and further Phase I–III randomized controlled trials are in progress to confirm or refute the role of immune checkpoint inhibition for NPC (8).

Aberrant cell cycle control is one of the common features of highly aggressive human malignancies (9), and dramatic accumulation of experimental and clinical evidence supports the notion that cell cycle machinery is commonly targeted on oncogenesis (10). The cell division cycle-associated (CDCA) gene family consists of eight members (CDCA1–8) that play an important role in mitosis, intersecting chromosome segregation and cell division with cancer. Cell division cycle-associated protein 1 (CDCA1), which is also named NUF1, plays an important role in kinetochore functions and the spindle checkpoint by binding with KNTC2 (11). Cell division cycle-associated protein 2 (CDCA2) can control the PP1γ-dependent DNA damage response in the cell cycle and preserve the characteristic chromosome architecture for the transition to interphase (12–14). Cell division cycle-associated protein 3 (CDCA3), which is referred to as a “trigger” of mitotic entry (15), mediates destruction of the mitosis-inhibitory kinase wee1 as a part of S phase kinase-associated protein 1/Cullin 1/F-box (SCF) E3 ubiquitin ligase complex (16). Cell division cycle-associated protein 4 (CDCA4) is involved in spindle organization from prometaphase and depletion of CDCA4 can result in accelerated cell proliferation (17). Cell division cycle-associated protein 5 (CDCA5) is an essential component for the stable binding of chromatids during S and G2/M phases and the maintenance of DNA strands’ stability during G2 phases (18, 19). Cell division cycle-associated protein 6 (CDCA6), also known as CBX2, is an essential regulator of gene expression and developmental programs (20). Cell division cycle-associated protein 7 (CDCA7) is reported as a c-Myc-responsive gene (21) and required for nucleosome remodeling by HELLS and for DNA methylation maintenance (22, 23). Cell division cycle-associated protein 8 (CDCA8) plays an indispensable role for transmission of the genome during cell division as a member of the chromosomal passenger complex (24). Much effort has been made in previous studies to demonstrate that the aberrant expression of CDCA gene family members play an indispensable role in tumorigenesis and are associated with the prognosis of many types of cancer (25–27). Notably, some studies have illuminated that CDCAs were associated with immune infiltration. Wang et al. (28) suggested that CDCAs show a powerful association with immune function regulation in hepatocellular carcinoma (HCC). Wu et al. (29) also indicated that CDCAs’ expression was correlated with the abundance of immune infiltrates in head and neck squamous cell carcinoma (HNSCC). These witnesses proved that abnormal expression of the CDCA gene family may modulate immune infiltration in the tumor microenvironment to promote oncogenesis. To the best of our knowledge, no studies have investigated the role and mechanism of the CDCA gene family in NPC prognosis. In this study, we found that the CDCA gene family was highly expressed in NPC by screening the differentially expressed genes in three pairs of NPC and nasopharyngitis tissues. Then, we found that CDCA8 was highly expressed in NPC and associated with prognosis by analyzing a clinical cohort with 130 patients. Additionally, we illuminated the clinical relevance and potential mechanism of CDCA gene family in multiple NPC cohorts by data mining. We aim to survey the connection between CDCA gene family and prognosis in NPC and explore the mechanism of CDCA gene family in NPC, providing a new thought for NPC therapy.

## Methods

### Sample Collection

Three pairs of NPC tissues and nasopharyngitis tissues were collected by endoscopic biopsy in the Affiliated Hospital of Guangdong Medical University. All tissues were confirmed by pathological examination and stored in liquid nitrogen immediately. A tissue microarray chip containing clinicopathological characteristics of 130 NPC patients was bought from the Shanghai Outdo Biotech Company. The experiment was approved by the ethics committee of the Affiliated Hospital of Guangdong Medical University, and all patients signed the consent for specimen retention. Two external cohorts, GSE102349 and GSE61218, were enrolled in this study, and the transcriptome profiles and clinical data were downloaded from the Gene Expression Omnibus (GEO) database (https://www.ncbi.nlm.nih.gov/geo/).

### Gene Chip Analysis

Total RNA of three pairs of tumor and non-tumor tissues was prepared with RNeasy Plus Mini Kit (Qiagen, Cat. No./ID: 74134). Gene chip was performed on the 4×180 k human ceRNA expression chip (Shanghai Bohao Tech Company, SBC human ceRNA array V1.0), which contained 68,423 lncRNAs and 18,853 mRNAs. The Low Input QuickAmp Labeling Kit (Agilent) was used to amplify and label the RNA, and the RNeasy Mini Kit (Qiagen) was used to clean up the cRNA after labeling. The hybrid reaction chamber was prepared with Gene Expression Hybridization Kit (Shanghai Bohao Tech Company) and the samples were hybrid in a rolling hybrid furnace with the following criteria: 65°C, 10 r/min. The Gene Expression Wash buffer Kit (Shanghai Bohao Tech Company) was used to wash the chip after hydrating for 17 h. Agilent Microarray and Feature Extraction software were used to scan and fetch origin data, respectively. The origin data were normalized by the R “limma” package. All experiments were performed according to the manufacturer’s protocols.

### Immunohistochemistry

Immunohistochemistry (IHC) was performed on the tissue microarray chip to determine the expression levels of CDCA8 protein. Antigen retrieval was performed by microwaving the sections in antigen retrieval solution (Maixin biotech, CHN) containing EDTA for 20 min and the sections were cooled in cold water. Then, the sections were washed with distilled water three times. Endogenous peroxidase activity was quenched using hydrogen peroxide solution for 10 min. Finally, the sections were washed by phosphate-buffered saline (PBS) three times. Bovine serum albumin (BSA; 5%) was used to seal the sections under 37°C for 30 min. Tissue sections were incubated with anti-human CDCA8 mouse biotinylated antibody at 1:150 (Novus Biologicals, Canada). After overnight incubation at 4°C, the sections were incubated at room temperature for 30 min and washed thrice in PBS for 5 min/time, and this was further incubated with the secondary antibody at 37°C for 30 min. After washing for three times and 5 min per time in PBS, the expression of CDCA8 was visualized with the development of diaminobenzidine (DAB) as a chromogen, and the sections were counterstained with hematoxylin, washed in tap water, dehydrated in graded alcohols, and mounted in xylene. Immunoreactivity of epithelial cells (cancer and normal) was scored based on a 4-point intensity scoring system (30): 0 = negative expression, 1 = weak positive, 2 = moderate positive, and 3 = strong positive. The ductal epithelium of the NPC tissues that were always stained positive served as the internal positive control for IHC.

### Gene Expression Analysis

DESeq2 (31) was applied to identify the differentially expressed CDCAs in our cohort (|FC| ≥ 1.5, *p* ≤ 0.01). As validation, comparison of CDCAs expression between NPC tissues and nasopharyngitis tissues was analyzed in the GSE61218 cohort. The log counts per million (logCPM) that transformed from raw counts by R “voom” package was used to measure the expression of genes.

### Prognosis Analysis

One hundred thirty tissues in the tissue microarray chip were divided into high- and low-expression groups based on the median value of IHC scores. As validation, 113 patients in GSE102349 were also divided into the high-expression group and low-expression group depending on the median expression of CDCAs. Kaplan–Meier survival analysis was used to compare the survival times between high- and low-expression groups and the log-rank test was used to test the significant difference.

### Differentially Expressed Gene Analysis and Functional Enrichment Analysis

Differentially expressed genes (DEGs) were identified by differentially expressed gene analysis using DESeq2 (31); the threshold value of differential gene analysis was false discovery rate (FDR) < 0.05 and |Fold Change (FC)| ≥ 1.5. Kyoto Encyclopedia of Genes and Genomes (KEGG) analyses were conducted using the R package “clusterProfiler” (32). Gene Set Enrichment Analysis (GSEA) (33) was conducted using the gseKEGG and gsePathway function in the R package “clusterProfiler”, with the following parameters: nPerm = 1000, minGSSize = 10, maxGSSize = 1000, and pvalue-Cutoff = 0.05.

### Immune Microenvironment Analysis

The xCell (34) tool was used to analyze the expression matrix to infer the cellular components in the immune microenvironment of NPC patients in GSE102349 and GSE61218. Immune checkpoint blockade (ICB)-related genes were collected (35). Pearson’s correlation analysis was used to evaluate the correlation between CDCAs and immune filtrate, and the correlation between CDCAs and ICB-related gene in GSE102349 and GSE61218. The *p*-value < 0.05 was considered as significant. To further confirm the important role of immune infiltrate in prognosis of NPC, NPC patients in GSE102349 were divided into a high infiltration group and a low infiltration group based on the median of each cell infiltration score. Kaplan–Meier survival analysis and log-rank test were used to compare the survival time between two groups.

### Statistical Analysis

Independent *t*-test was used to compare normally distributed continuous variables and Mann–Whitney *U* test was used to compare skewed continuous variables. Two-tailed *p* < 0.05 was used to determine statistical significance. All the statistical analysis and visualization were performed with the R version 4.0.2 (Institute for Statistics and Mathematics, Vienna, Austria 4). The flow chart (36) of this study is shown as **Supplementary Figure 1**.

## Results

### CDCAs Were Highly Expressed in NPC

**Figure 1:**
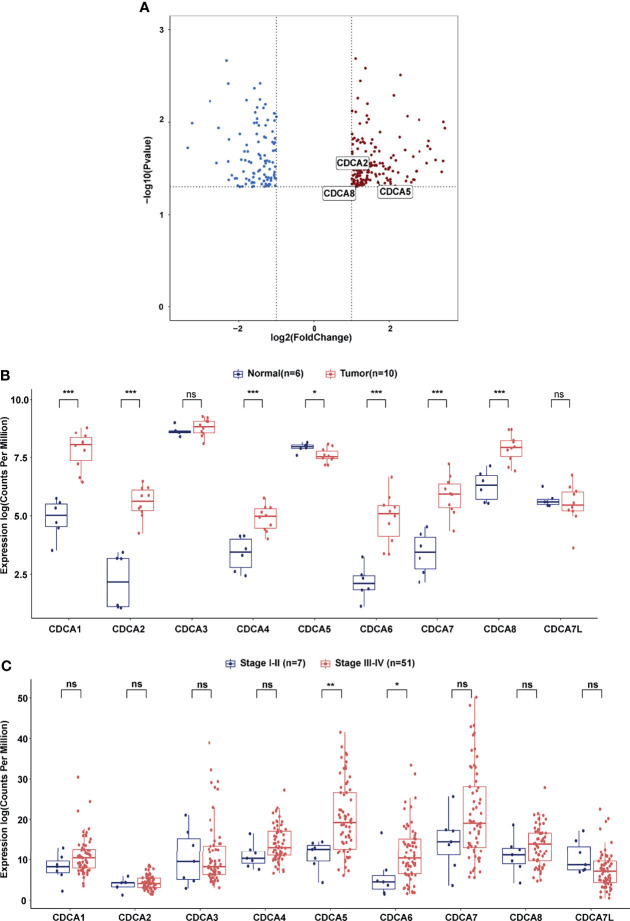
CDCA gene family members expression analysis. **(A)** Volcano plot of differentially expressed genes between NPC samples and nasopharyngitis samples. The blue dot represents downregulated genes and the red dot represents upregulated genes. **(B)** Differential expression of CDCA gene family members between NPC tissues and nasopharyngitis tissues in GSE61218. **(C)** Differential expression of CDCA gene family members between NPC patients in stage I–II and NPC patients in stage III–IV in GSE102349. “*” represents *p* < 0.05, “**” represents *p* < 0.01, “***” represents *p* < 0.001, “ns” represents no significant difference.

Gene chip analysis showed that 184 upregulated genes and 106 downregulated genes were identified as DEGs. Notably, three members of the CDCA gene family, CDCA2 (FC = 2.21, *p* = 0.038), CDCA5 (FC = 3.10, *p* = 0.036), and CDCA8 (FC =2.17, *p* = 0.039), were found to be upregulated in NPC tissues compared with the NE tissues (**Figure 1A**, **Supplementary Table 1**). Then, we compared the expression of CDCAs between NPC and NE tissue specimens in GSE61218. The expression of CDCA1/2/4/6/7/8 was higher in NPC tissues than in nasopharyngitis tissues (**Figure 1B**, all *p* < 0.001). Subsequently, we stratified the NPC patients in GSE102349 based on the tumor stage. NPC patients in stage I–II had lower expression of CDCA5 (*p* < 0.01) and CDCA6 (*p* < 0.05) than those in stage III–IV. No significant difference was found in CDCA1/2/3/4/7/8/7L (**Figure 1C**).

### High Expression of CDCA3/5/8 Was Associated With Poor Prognosis in NPC

**Figure 2:**
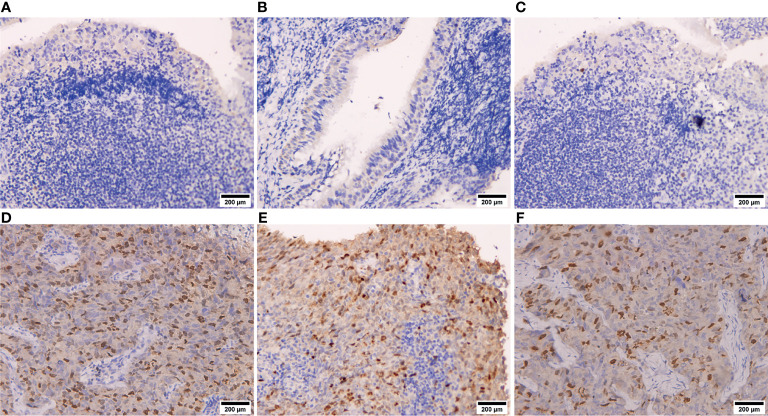
Immunohistochemistry of NPC tissues and nasopharyngitis tissues. **(A–C)** CDCA8 protein expression in nasopharyngitis tissues. **(D–F)** CDCA8 protein expression in NPC tissues.

**Figure 3:**
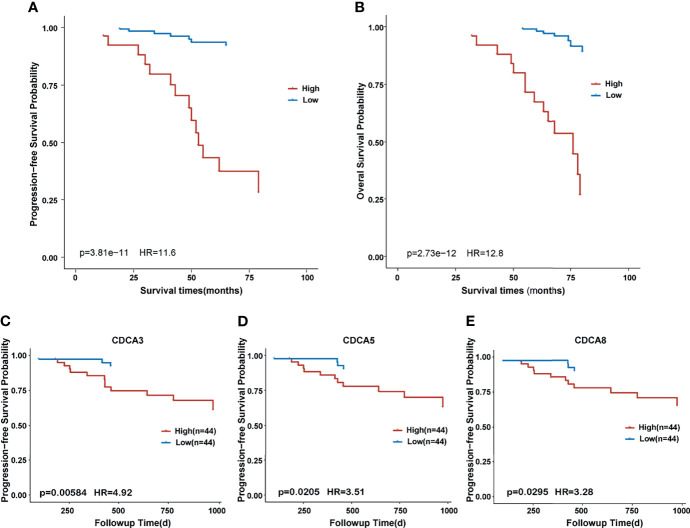
Kaplan–Meier survival curve between different expression groups of CDCA gene family members. **(A)** Survival curve of progression-free survival in high and low expression of CDCA8 in our cohort. **(B)** Survival curve of overall survival in high and low expression of CDCA8 in our cohort. **(C)** Survival curve of progression-free survival in high and low expression of CDCA3 in the GSE102349 cohort. **(D)** Survival curve of progression-free survival in high and low expression of CDCA5 in the GSE102349 cohort. **(E)** Survival curve of progression-free survival in high and low expression of CDCA8 in the GSE102349 cohort.

**Figure 4:**
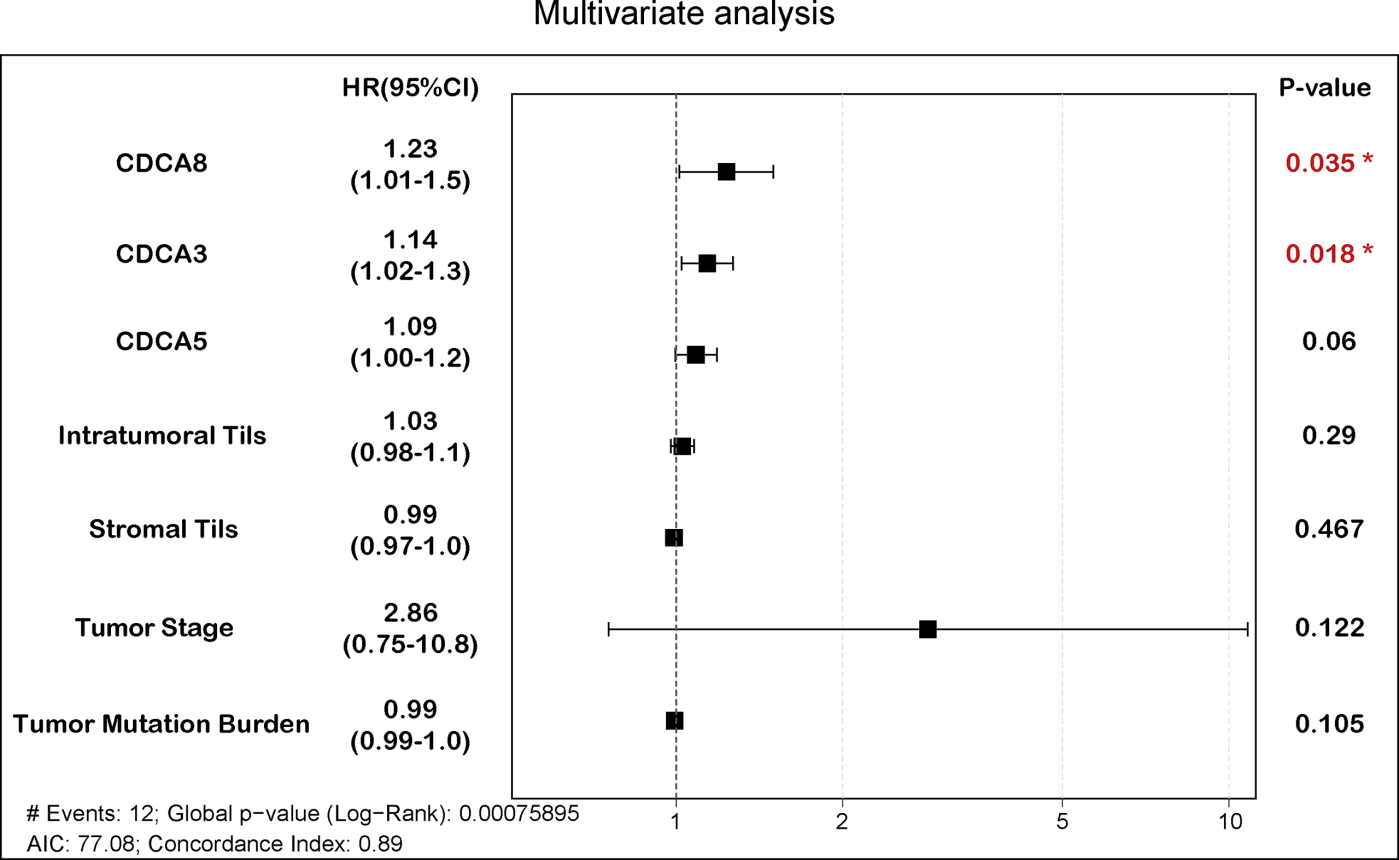
Multivariate analysis of CDCA3, CDCA5, CDCA8, and clinical features. The boxplot represents hazard ratios and confidence intervals. “*” represents *p*value < 0.05.

As shown in **Figure 2**, IHC suggested that CDCA8 was highly expressed in NPC tissues and the expression of CDCA8 was very low in nasopharyngitis tissues. Then, 130 patients in our cohort were divided into high- and low-expression groups based on the median value of the IHC scores. Comparison of the clinical features between the two groups showed that patients with N1–N3 were gathered more in the high CDCA8 expression group (*p* < 0.001) while no significant difference was found between the two groups in other clinical features (**Table 1**). Kaplan–Meier curves suggested that high expression of CDCA8 indicated poor progression-free survival (PFS) (**Figure 3A**, *p* < 0.001) and overall survival (OS) (**Figure 3B**, *p* < 0.001). As validation, prognosis analysis was also performed in the GSE102349 dataset. In GSE102349, NPC patients with high expression of CDCA3 (**Figure 3C**, *p* = 0.00584), CDCA5 (**Figure 3D**, *p* = 0.0205), or CDCA8 (**Figure 3E**, *p* = 0.0295) had shorter PFS, while no significant difference was found in other CDCA gene family members (**Supplementary Figure 2**). Afterwards, comparison of clinical characteristics between patients with high and low expression of CDCA3, CDCA5, and CDCA8 was performed in GSE61218 and GSE102349. However, no significant difference was found in both cohorts (**Supplementary Table 2**). Multivariate analysis was performed in GSE102349 and the result showed that CDCA8 (HR = 1.23, 95% CI = 1.01–1.5) and CDCA3 (HR = 1.14, 95% CI = 1.02–1.3) were independent risk factors for prognosis in NPC (**Figure 4**, *p* < 0.05).

**Table 1:** Clinical characteristics between high and low CDCA8 expression groups.

Characteristics	Overall/*n* (%)	Frequency in low CDCA8 expression group (%)	Frequency in high CDCA8 expression group (%)	*p*-value*
Gender				0.392
Male	99 (76.2)	29 (82.9)	70 (73.7)	
Female	31 (23.8)	6 (17.1)	25 (26.3)	
Age				0.087
<60 years	107 (82.3)	25 (71.4)	82 (86.3)	
≥60 years	23 (17.7)	10 (28.6)	13 (13.7)	
Progression				0.147
No progression	70 (53.8)	23 (65.7)	47 (49.5)	
Progression	60 (46.2)	12 (34.3)	48 (50.5)	
Tumor Stage				0.082
I–II	71 (54.6)	24 (68.6)	47 (49.5)	
III–IV	59 (45.4)	11 (31.4)	48 (50.5)	
Status				0.535
Alive	109 (83.8)	31 (88.6)	78 (82.1)	
Death	21 (16.2)	4 (11.4)	17 (17.9)	
T				0.272
T1–T2	81 (62.3)	25 (71.4)	56 (58.9)	
T3–T4	49 (37.7)	10 (28.6)	39 (41.1)	
M				0.509
M0	126 (96.9)	35 (100)	91 (95.8)	
M1	4 (3.1)	0 (0)	4 (4.2)	
N				<0.001
N0	49 (37.7)	28 (80)	21 (22.1)	
N1–N3	81 (62.3)	7 (20)	74 (77.9)	

*Chi-square test, p-value < 0.05 means significantly different.

### CDCA8 Was Associated With Immune-Related Pathway in NPC

**Figure 5:**
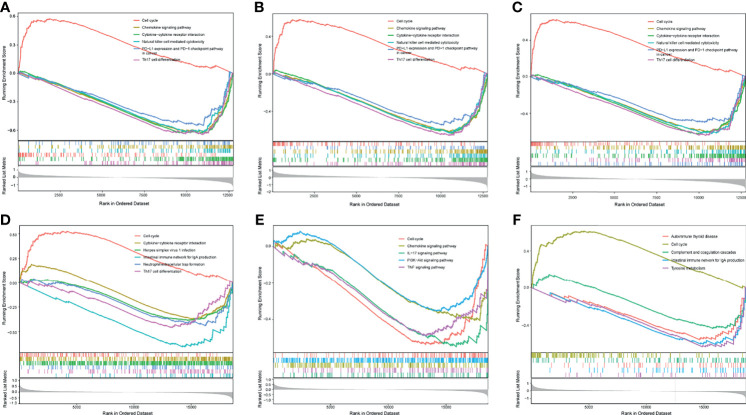
GSEA analysis of differentially expressed genes (DEGs). **(A)** GSEA analysis of DEGs between high and low expression of CDCA3 groups in GSE102349. **(B)** GSEA analysis of DEGs between high and low expression of CDCA5 groups in GSE102349. **(C)** GSEA analysis of DEGs between high and low expression of CDCA8 groups in GSE102349. **(D)** GSEA analysis of DEGs between high and low expression of CDCA3 groups in GSE61218. **(E)** GSEA analysis of DEGs between high and low expression of CDCA5 groups in GSE61218. **(F)** GSEA analysis of DEGs between high and low expression of CDCA8 groups in GSE61218.

In GSE102349, overexpression of CDCA3, CDCA5, and CDCA8 was significantly associated with the activation of the cell cycle pathway, inversely, and these three genes’ overexpression was also related to the inactivation of some immune-related pathways, such as Chemokine signaling pathway and PD-L1/PD-1 checkpoint pathway (**Figures 5A–C**). Likewise, in GSE61218, high expression of CDCA3 and CDCA8 was associated with the activation of cell cycle pathway. Besides, overexpression of CDCA3 and CDCA8 was related to downregulation of some immune-related pathways, including Chemokine signaling pathway and Th17 cell differentiation (**Figures 5D, F**). However, the expression of CDCA5 was associated with downregulation of cell cycle genes set and some immune pathways genes sets in GSE61218 (**Figure 5E**). The possible reason for this result was the small number of patients in the GSE12618.

### High Expression of CDCA8 Indicated Poor Immune Infiltrate and Immunotherapy Response in NPC

**Figure 6:**
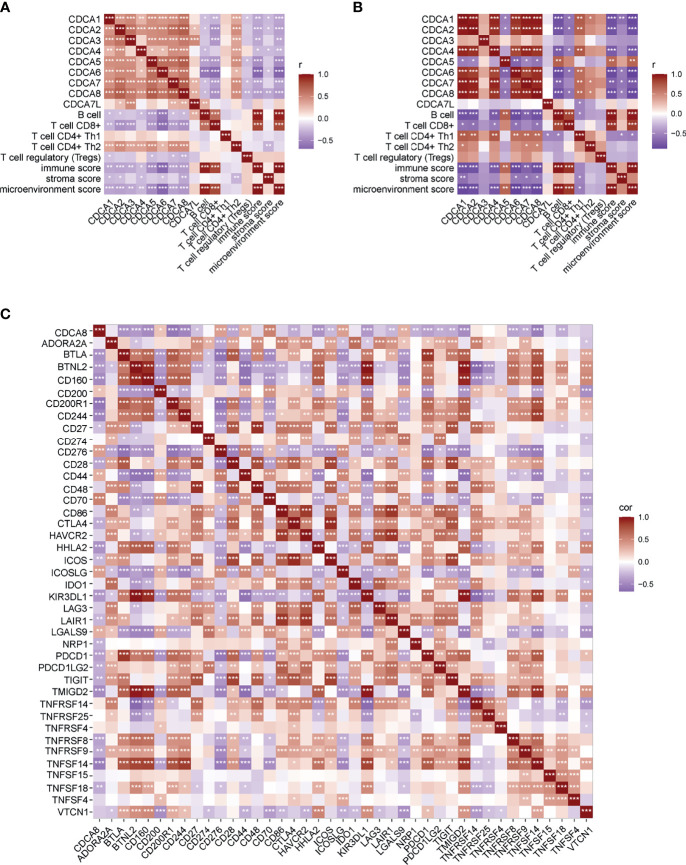
Correlation of CDCA gene family members and immune infiltration and ICB-related genes. **(A)** Correlation of CDCA gene family members and immune infiltration in GSE102349. **(B)** Correlation of CDCA gene family members and immune infiltration in GSE61218. **(C)** Correlation of CDCA gene family members and ICB-related genes in the dataset combined by GSE102349 and GSE61218. *p < 0.05, **p < 0.01, and ***p < 0.001.

**Figure 7:**
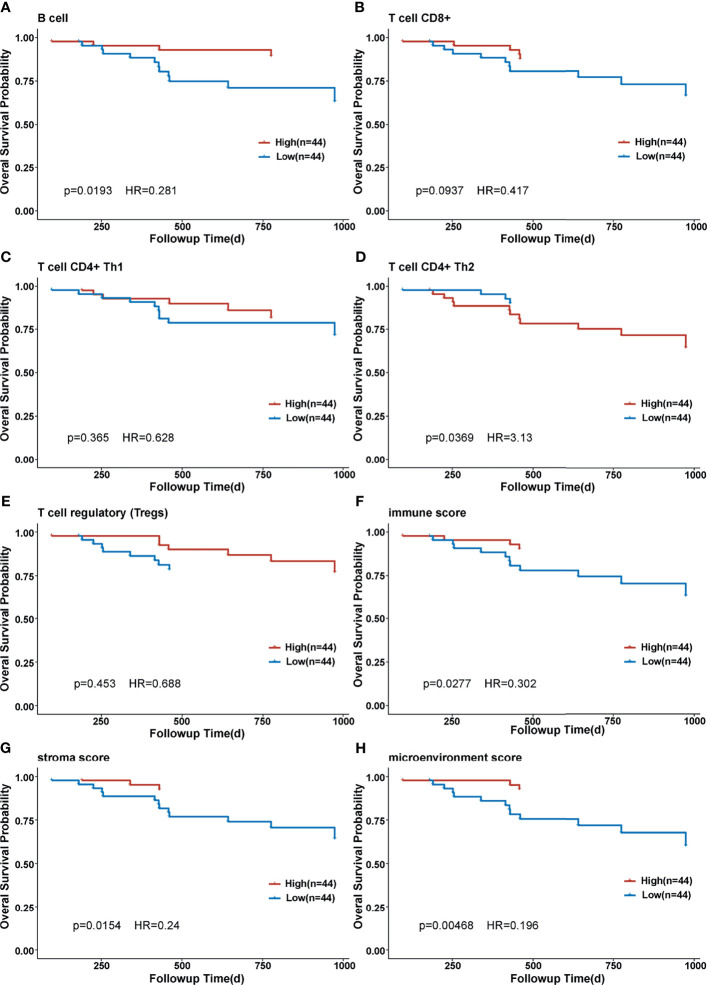
Kaplan–Meier curve of overall survival in different immune cell-infiltrated groups. **(A)** Kaplan–Meier curve of overall survival in different B-cell-infiltrated groups. **(B)** Kaplan–Meier curve of overall survival in different CD8^+^ T-cell-infiltrated groups. **(C)** Kaplan–Meier curve of overall survival in different CD4^+^ Th1-cell-infiltrated groups. **(D)** Kaplan–Meier curve of overall survival in different CD4^+^ Th2-cell-infiltrated groups. **(E)** Kaplan–Meier curve of overall survival in different T cell regulatory-infiltrated groups. **(F)** Kaplan–Meier curve of overall survival in different immune score groups. **(G)** Kaplan–Meier curve of overall survival in different stroma score groups. **(H)** Kaplan–Meier curve of overall survival in different microenvironment score groups.

Correlation of CDCA gene family members and immune infiltration was performed in two datasets, separately. In GSE102349, overexpression of CDCA gene family members was associated with less infiltration of B cell, CD8+ T cell, and Tregs cell, and lower immune score, stromal score, and microenvironment score. On the contrary, CDCA gene family members’ overexpression indicated more CD4+ Th1 and CD4+ Th2 infiltration (**Figure 6A**). In GSE61218, most of the CDCA gene family members were negatively correlated with B cell, CD8+ T cell, immune score, stromal score, and microenvironment score while CDCA5 displayed the opposite condition (**Figure 6B**). The possible reason for this result was the small number of patients in GSE12618. Subsequently, we evaluated the correlation between CDCA8 and ICB-related genes in the dataset combined by GSE102349 and GSE61218. As displayed by the heatmap, the CDCA8 expression was negatively correlated with the expression of multiple ICB-related genes (**Figure 6C**). Eleven of the most relevant ICB-related genes were CD200R1, TNFSF14, CD27, CD244, CD160, TMIGD2, BTLA, HHLA2, KIR3DL1, BTNL2, and PDCD1. Besides, CDCA8 was mostly positively correlated with CD70. Then, NPC patients were divided into high- and low-infiltration groups based on the median cell infiltration score of each type, and the survival difference between the two groups was compared. The high infiltration group of B cells was conducive to survival (**Figure 7A**, *p* = 0.0193), while high infiltration of CD4+ Th2 was associated with inferior prognosis (**Figure 7D**, *p* = 0.0369). Besides, patients with the higher immune score, stroma score, and microenvironment score had significantly longer OS than those with low scores (**Figures 7F–H**, all *p* < 0.05). The evidence suggested that the CDCA gene family may impact prognosis by involving the immunoregulation of NPC.

## Discussion

Cancers are propelled by a limited number of critical events into an uncontrolled invasion. Deregulated cell proliferation is one of the critical events and provides a minimal platform necessary to support further neoplastic progression together with the obligate compensatory suppression of apoptosis (37). In recent years, numerous studies have illustrated cell cycle control as a promising target in cancers (38–40). The cell cycle kinase inhibitors have been developed and studies on the role of cell cycle inhibitors in cancers are ongoing (41, 42). Cell cycle regulation depends on phase-specific transcriptions of cell cycle genes, which included the cell division cycle-associated gene family (CDCAs). The deregulation of CDCAs has been reported in many cancers, including ovarian cancer (43), breast cancer (44), and lung carcinoma (45). However, the role of CDCAs in NPC has not been investigated yet. In this study, the expression of CDCAs in NPC was evaluated by gene chip and validated in GSE61218. The result indicated that CDCAs were significantly upregulated in NPC. Then, the prognostic value of CDCAs in NPC was evaluated in an IHC microarray and validated in GSE102349. Kaplan–Meier survival analysis in GSE102349 indicated that high expression of CDCA3/5/8 was associated with poor prognosis in NPC. Our findings indicated that CDCAs were upregulated and associated with prognosis in NPC, providing new insight into CDCAs in NPC.

To further reveal the potential mechanism of CDCAs in NPC, GSEA analysis was performed. The results showed that CDCA3, CDCA5, and CDCA8 were associated with cell cycle and some immune-related pathways, indicating that CDCA gene family members might participate in the immunoregulation of NPC. Therefore, we analyzed the correlation between CDCA gene family members and immune infiltration in NPC. CDCA gene family members were negatively correlated with CD8+ T cell, immune score, stromal score, and microenvironment score strongly. CD8+T cell is a kind of specific T cell that plays an important anti-tumor role. CD8+ T-cell differentiation and the infiltration of CD8+ T cells into the tumor microenvironment (TME) are two crucial basic conditions for the anti-tumor role of CD8+ T cells (44). Researchers have reported a positive correlation between elevated CD8+ T cells in the TME and a good prognosis in cancer (45). Our findings suggested that the overexpression of CDCAs may prevent the infiltration of CD8+ T cells into TME, resulting in poor outcomes in NPC patients. In the functional enrichment analysis, we also suggested that CDCA8 was associated with the PD-1/PD-L1 signaling pathway. Therefore, we evaluated the correlation of CDCA8 and ICB-related genes, and the result showed that CDCA8 was negatively correlated with some important ICB-related genes including HHLA2, CTLA4, and CD244. HERV-H LTR-Associating 2 (HHLA2) is a member of the B7-CD28 family, which regulates T-cell function (46). An increasing number of studies have demonstrated HHLA2 as a new immune checkpoint molecule and prognostic biomarker in cancers, especially in PD-1-negative human tumors (47–49). Cytotoxic T-Lymphocyte-Associated Protein 4 (CTLA4) and CD28 are members of the immunoglobulin superfamily. The function of CTLA4 in tumorigenesis and tumor immunity has been widely investigated (50), and some CTLA-4 inhibitors were clinically actionable. Our findings indicated that CDCA8 was associated with ICB-related genes and might be a potentially reliable biomarker for the efficacy of ICB therapy. CD244 is an immunomodulatory transmembrane receptor in the signaling lymphocyte activation molecule family. CD244 expression was increased in head and neck squamous cell carcinoma (HNSCC) and negatively correlated with CD8+ T-cell infiltrate, suggesting that CD244 could be an immunotherapy target in HNSCC (51). Our findings suggested that CDCA8 could be the potential predictor for immunotherapy response in NPC.

This study provided new insight into CDCA gene family members in NPC, and the prognostic signature based on CDCA3, CDCA5, and CDCA8 may facilitate clinicians to identify more aggressive and immunosuppressive tumors and make more individually appropriate therapeutic decisions. Moreover, we also offered a potentially new perspective for identifying prognostic biomarkers of immunotherapy in NPC. However, there are some limitations of this study. For example, validating the biological significance of CDCA genes with *in vitro* and *in vivo* experiments would be advantageous. Besides, the immune function of CDCA gene family members in NPC has not been fully explained and still needs to be further investigated. In the future, we will attempt to work on the shortcomings.

## Conclusion

Overexpression of CDCA3, CDCA5, and CDCA8 indicated poor prognosis in NPC. High expression of CDCA8 was associated with poor immune infiltration and negatively correlated with ICB-related genes. Our findings might shed light on novel targets for treatment strategies.

## Data Availability Statement

The datasets presented in this study can be found in online repositories. The names of the repository/repositories and accession number(s) can be found in the article/**Supplementary Material**.

## Ethics Statement

The studies involving human participants were reviewed and approved by the Affiliated Hospital of Guangdong Medical University. The patients/participants provided their written informed consent to participate in this study. Written informed consent was not obtained from the individual(s) for the publication of any potentially identifiable images or data included in this article.

## Author Contributions

Study concept and design: DJ and MX. Acquisition of materials and data: DJ and MX. Analysis and interpretation of data: DJ, YL, JC, LS, XZ, and MX. Drafting of the manuscript: DJ. Statistical analysis: DJ. Administrative, technical, or material support: DJ, YL, JC, LS, XZ, and MX. Supervision: MX. All authors contributed to the article and approved the submitted version.

## Conflict of Interest

The authors declare that the research was conducted in the absence of any commercial or financial relationships that could be construed as a potential conflict of interest.

## Publisher’s Note

All claims expressed in this article are solely those of the authors and do not necessarily represent those of their affiliated organizations, or those of the publisher, the editors and the reviewers. Any product that may be evaluated in this article, or claim that may be made by its manufacturer, is not guaranteed or endorsed by the publisher.

## Supplementary Material

Supplementary Figure 1 The flow chart of this study.

Supplementary Figure 2 Kaplan-Meier survival curves between the high expression group and the low expression group of CDCA1 **(A)**, CDCA2 **(B)**, CDCA4 **(C)**, CDCA6 **(D)**, CDCA7 **(E)**, CDCA7L **(F)**, respectively.
