# Supplementary figures and images for: Cell Division Cycle-Associated Genes Are Potential Immune Regulators in Nasopharyngeal Carcinoma
Source: Front Oncol. 2022 Feb 14;12:779175. doi: 10.3389/fonc.2022.779175 (PMC8882974; doi:10.3389/fonc.2022.779175)

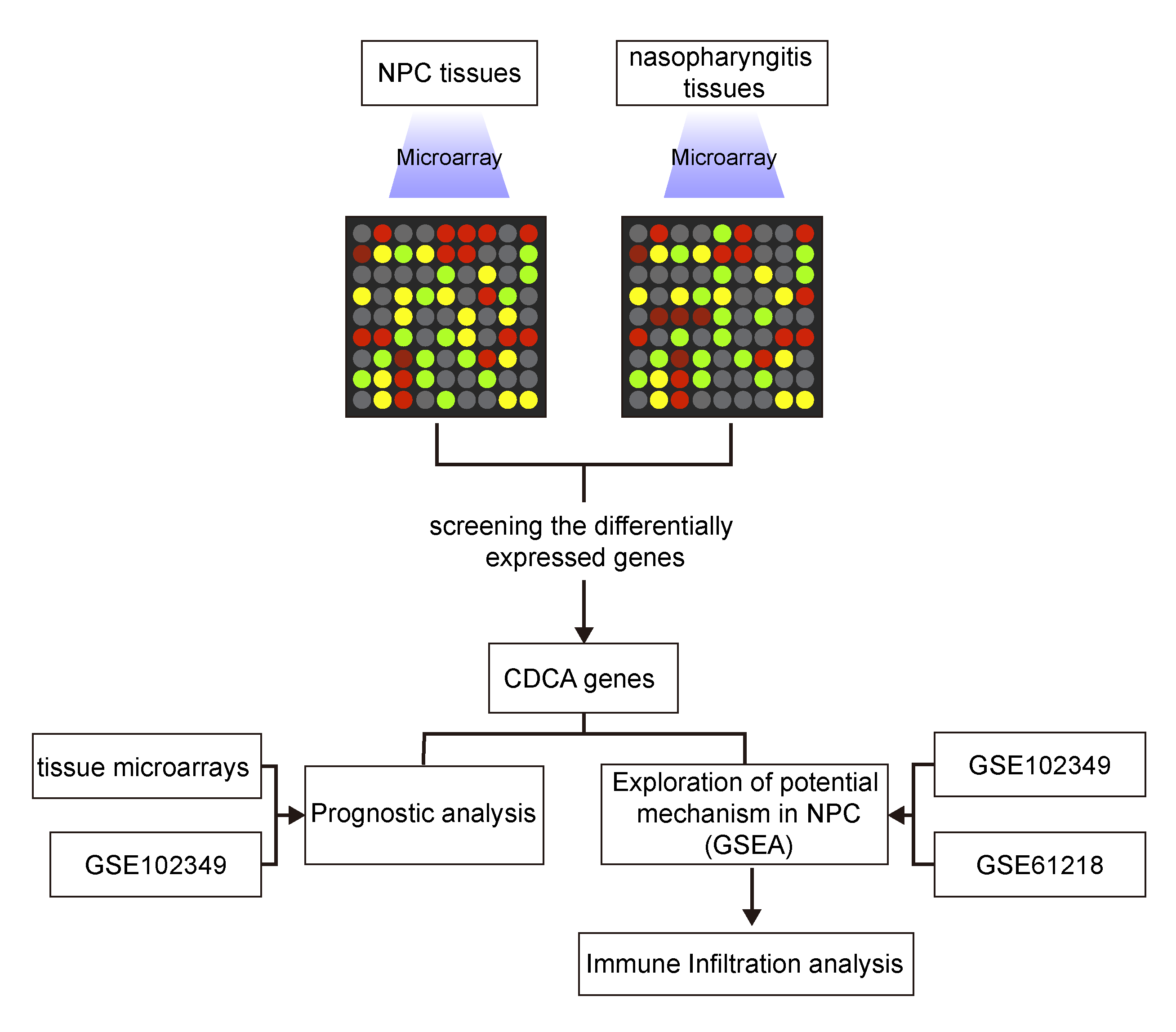

Supplement: Supplementary Figure 1 — The flow chart of this study. [file Image_1.tif]

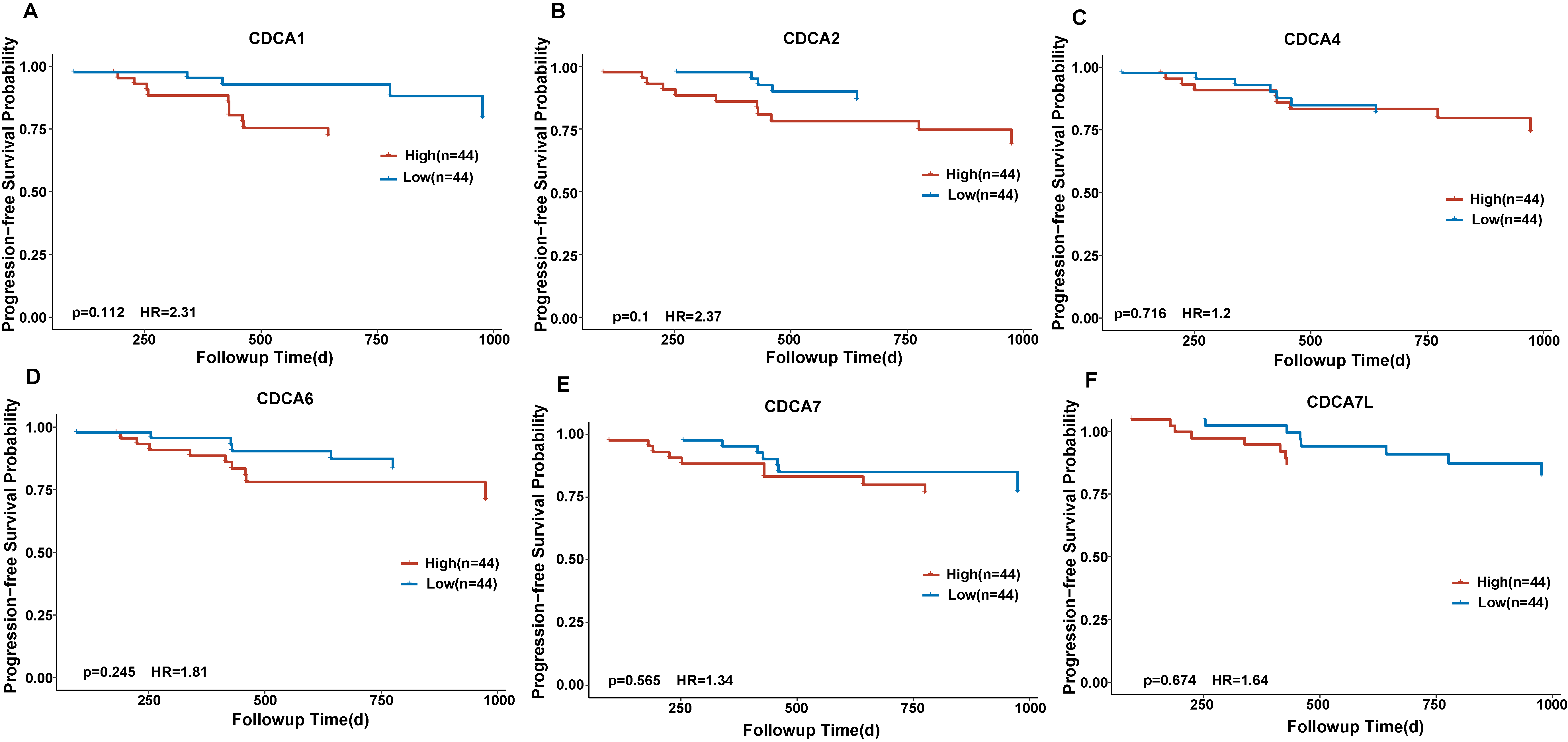

Supplement: Supplementary Figure 2 — Kaplan-Meier survival curves between the high expression group and the low expression group of CDCA1 (A), CDCA2 (B), CDCA4 (C), CDCA6 (D), CDCA7 (E), CDCA7L (F), respectively. [file Image_2.tif]
